# Congenital Hypopituitarism During the Neonatal Period: Epidemiology, Pathogenesis, Therapeutic Options, and Outcome

**DOI:** 10.3389/fped.2020.600962

**Published:** 2021-02-02

**Authors:** Laura Bosch i Ara, Harshini Katugampola, Mehul T. Dattani

**Affiliations:** ^1^Department of Paediatric Endocrinology, Great Ormond Street Hospital for Children, London, United Kingdom; ^2^Genetics and Genomic Medicine Programme, UCL Great Ormond Street Institute of Child Health, London, United Kingdom

**Keywords:** hypopituitarism, newborn, hypoglycaemia, pituitary gland, hormone deficiencies, septo-optic dysplasia, growth hormone, micropenis

## Abstract

**Introduction:** Congenital hypopituitarism (CH) is characterized by a deficiency of one or more pituitary hormones. The pituitary gland is a central regulator of growth, metabolism, and reproduction. The anterior pituitary produces and secretes growth hormone (GH), adrenocorticotropic hormone, thyroid-stimulating hormone, follicle-stimulating hormone, luteinizing hormone, and prolactin. The posterior pituitary hormone secretes antidiuretic hormone and oxytocin.

**Epidemiology:** The incidence is 1 in 4,000–1 in 10,000. The majority of CH cases are sporadic; however, a small number of familial cases have been identified. In the latter, a molecular basis has frequently been identified. Between 80–90% of CH cases remain unsolved in terms of molecular genetics.

**Pathogenesis:** Several transcription factors and signaling molecules are involved in the development of the pituitary gland. Mutations in any of these genes may result in CH including *HESX1, PROP1, POU1F1, LHX3, LHX4, SOX2, SOX3, OTX2, PAX6, FGFR1, GLI2*, and *FGF8*. Over the last 5 years, several novel genes have been identified in association with CH, but it is likely that many genes remain to be identified, as the majority of patients with CH do not have an identified mutation.

**Clinical manifestations:** Genotype-phenotype correlations are difficult to establish. There is a high phenotypic variability associated with different genetic mutations. The clinical spectrum includes severe midline developmental disorders, hypopituitarism (in isolation or combined with other congenital abnormalities), and isolated hormone deficiencies.

**Diagnosis and treatment:** Key investigations include MRI and baseline and dynamic pituitary function tests. However, dynamic tests of GH secretion cannot be performed in the neonatal period, and a diagnosis of GH deficiency may be based on auxology, MRI findings, and low growth factor concentrations. Once a hormone deficit is confirmed, hormone replacement should be started. If onset is acute with hypoglycaemia, cortisol deficiency should be excluded, and if identified this should be rapidly treated, as should TSH deficiency. This review aims to give an overview of CH including management of this complex condition.

## Introduction

Congenital hypopituitarism (CH) is defined as the deficiency of one or more hormones produced by the anterior pituitary (AP) or released from the posterior pituitary (PP). Its estimated incidence is between 1 in 4,000 and 1 in 10,000 live births (1).

The pituitary gland is the central regulator of growth, metabolism, reproduction and homeostasis. It is located in the midline of the brain within the sella turcica and consists of three lobes of dual embryologic origin. The adenohypophysis (anterior and intermediate lobes) originates from Rathke's pouch, an invagination of the oral ectoderm, whereas the neurohypophysis (posterior lobe) develops from the neural ectoderm of the ventral diencephalon.

The AP consists of five different cell lineages producing six hormones: somatotrophs (growth hormone, GH), gonadotrophs (follicle stimulating hormone, FSH, and luteinising hormone, LH), corticotrophs (adrenocorticotropic hormone, ACTH), thyrotrophs (thyroid stimulating hormone, TSH), and lactotrophs (prolactin, PRL). The intermediate lobe contains melanotrophs, which secrete proopiomelanocortin (POMC), a major precursor to endorphins, and melanocyte-stimulating hormone (MSH). The PP lobe releases two hormones, oxytocin and antidiuretic hormone (ADH, also known as vasopressin), which are produced in the hypothalamus (supraoptic and paraventricular nuclei) and transported axonally via the pituitary stalk to be stored and released from the PP.

The hypothalamic parvocellular neurosecretory system is responsible for the release of specific AP hormones. It consists of neurons secreting thyrotrophin-releasing hormone (TRH) stimulating secretion of TSH and PRL, corticotrophin-releasing hormone (CRH) that acts to stimulate the secretion of ACTH, gonadotrophin releasing hormone (GnRH) that stimulates release of FSH and LH, growth hormone releasing hormone (GHRH) that stimulates the secretion of GH, somatostatin (SS) that negatively regulates GH secretion, and dopamine that inhibits secretion of PRL. These hypothalamic factors are rapidly transported to the AP via the hypophyseal portal blood system (2, 3).

The aim of this review is to describe the range of mechanisms underlying CH, clinical findings during the neonatal period, diagnosis, treatment, and future therapeutic options.

## Etiology

CH may occur due to developmental defects of the pituitary gland, in some cases as a result of genetic mutations. Acquired forms of hypopituitarism, secondary to perinatal or neonatal events, rarely occur in the neonatal period. CH may present as isolated or combined pituitary hormone deficiencies (CPHD), and may be part of a syndrome involving extra-pituitary abnormalities.

In the majority of cases, the etiology of CH is unknown. The overall incidence of genetic mutations in these patients is low (16% of cases can currently be explained by mutations in known genes) indicating that many genes remain to be identified (4). *PROP1* mutations are the most frequent known cause of both familial and sporadic congenital CPHD. Mutations in other genes have also been described, but appear to be much rarer. However, it is likely during the next few years that novel genetic determinants of pituitary disorders will probably be identified with the availability of next generation sequencing technology (5).

## Embryology and Genetics

The development of the pituitary gland is a multifactorial process that results from the temporo-spatial interactions of transcription factors and signaling molecules. These occur in distinct and sequential developmental steps. Although direct evidence in humans is lacking, the process of pituitary development is highly conserved across all vertebrate species including rodents, and development of the mouse pituitary, in particular, is well-characterized (6).

Pituitary gland development starts at an equivalent human gestational age of 4–6 weeks and occurs in 4 stages: (i) the pituitary placode, (ii) the rudimentary Rathke's pouch, (iii) the definitive Rathke's pouch, and (iv) the mature pituitary gland.

In the mouse the pituitary placode appears at embryonic (E) day 7.5, located ventrally to the anterior neural ridge and next to the future hypothalamo-infundibular region, which will give rise to the roof of the oral cavity. Initial pituitary development consists of a thickening of the roof of the oral ectoderm at E8.5. By E9.0 it invaginates dorsally and the rudimentary Rathke's pouch is formed (7). The definitive Rathke's pouch, formed by E10.5, gives rise to the anterior and intermediate lobes. The posterior lobe is derived from the posterior part of the developing diencephalon. By E12.5, the precursors of the hormone-secreting cells start to proliferate ventrally from the pouch to constitute the future AP.

Normal pituitary organogenesis requires apposition between the rudimentary Rathke's pouch and the diencephalon. This is critical as the induction and correct formation of the pouch requires at least two sequential inductive signals from the diencephalon (8, 9). Bone Morphogenetic Protein 4 (Bmp4) is the first secreted signaling molecule. Fibroblast Growth Factor 8 (Fgf8) that is the second signal activates two key regulatory genes, LIM homeobox 3 (*Lhx3*) and LIM homeobox 4 (*Lhx4*) that play a critical role in the development of the rudimentary pouch into a definitive pouch (10–12).

These signaling molecules are derived from different embryogenic origins: the ventral diencephalon (Bmp4, Fgf8, Fgf4, Nkx2.1, Wnt5α), the oral ectoderm (Sonic Hedgehog, Shh, the surrounding mesenchyme (Bmp2, Chordin) and the pouch (Bmp2, Wnt4) (13, 14).

Transcription factors expressed early in pituitary organogenesis include *Hesx1, Lhx3, Lhx4, Sox2, Sox3, Gli2*, and *Otx2*. *Prop1* and *Pou1f1* (previously known as *Pit1*) are implicated in the later stages.

Further cell determination and specification rely on the expression and interaction of multiple signaling molecules and transcription factors (15) and these will be expanded upon in the following section.

## Correlation Between Phenotype-Genotype

The variety of phenotypes seen in patients with CPHD is a reflection of the close developmental relationships seen during organogenesis of the pituitary gland, eye, optic nerve, ear, nose, and cranial nerve ganglia.

As a general rule, genetic mutations in genes involved in early development (*HESX1, LHX3, LHX4, SOX2, SOX3, GLI2*, and *OTX2*) tend to be part of a syndrome that includes extra-pituitary defects and midline abnormalities such as cleft lip and/or palate, as well as CH (Table 1). In contrast, mutations in the genes implicated in later stages (*PROP1* and *POU1F1*) result in variable phenotypes of CPHD without any extra-pituitary defects (16) (Table 2).

**Table 1:** Mutations and characteristics of genes involved in syndromic hypopituitarism.

	**Transcription factor**	**Inheritance**	**Hormone deficiencies**	**MRI**	**Phenotype**
**Septo-optic dysplasia and its variants**	*HESX1*	AR, AD	Isolated GHD CHPD	APH EPP ACC	SOD and its variants
	*SOX2*	AD	LH, FSH deficiency Variable GHD	APH Thin corpus callosum Hippocampal abnormalities Hypothalamic hamartoma Slow progressing hypothalamo-pituitarytumour	Anophthalmia/microphthalmia Esophageal atresia Genital tract abnormalities Sensorineural hearing loss Hypothalamic hamartoma Spastic diplegia Mental retardation Dentalanomalies
	*SOX3*	X-linked	Pan hypopituitarism GH, TSH, ACTH, LH, and FSH deficiencies Isolated GHD	APH EPP Absent pituitary stalk Persistent craniopharyngeal canal Infundibularhypoplasia	Mental retardation Craniofacial abnormalities Hearing impairment
	*OTX2*	AD	Isolated GHD CPHD (GH, TSH, PRL, LH, FSH deficiencies)	Normal APH EPP Chiari Syndrome	Eye malformations (bilateral anophthalmia or severe microphthalmia, or coloboma) Developmental delay Seizures
	*PAX 6*	AR	GHD ACTH deficiency FSH, LH deficiency	APH	Eye malformations
	*BMP4*	AR	CPHD	Cerebellar abnormalities Partial ACC	Eye malformations Sensorineural hearing loss Developmental delay Spondyloepiphyseal dysplasiatarda
	*FGFR1*	AD	CPHD (GH, TSH, ACTH, LH, FSH deficiency) with DI	APH EPP Stalk thin or normal ACC	SOD Eye malformations Cleft lip/palate Brachydactyly Central incisor Kallman Syndrome
	*ARNT2*	AR	DI, ACTH, GH, and TSH deficiencies CPHD	APH Absent PP Stalk thin ACC Frontal and temporal lobe hypoplasia Large Sylvian fissure	Eye malformations Microcephaly Renal abnormalities Seizures
**Holoprosencephaly**	*GLI2*	ADHaplo-insufficiency	CPHD GH, TSH, ACTH, LH, and FSH deficiencies Isolated GHD	Holoprosencephaly APH EPP or normal PP Hypoplastic corpus callosum Cavum septumpellucidum	Midfacial defects Cleft lip/palate Single central incisor Postaxial polydactyly ONH
	*FGF8*	ADAR	Hypopituitarism LH, FSH deficiencies TSH deficiency ACTH deficiency DI Borderline peak GH concentration	Holoprosencephaly ACC ONH	Kallman Syndrome (HH + Anosmia) Moebius syndrome Spastic diplegia Developmental delay SOD
**PSIS**	*OTX2*	AD	IGHD CPHD (GH, TSH, PRL, LH, FSH deficiencies)	Normal APH EPP Chiari syndrome	Anophthalmia (bilateral/unilateral) Coloboma Retinal dystrophy Normal eye phenotype Bilateral severe microphthalmia Seizures Developmental delay
	*PROKR2*	ADAR	GH, TSH, ACTH, LH, FSH deficiencies GHD	PSIS APH or normal AP EPP Absent stalk Dysgenesis of corpus callosum Other: Schizencephaly Cerebellar hypoplasia Hypoplastic optic discs and nerves	Neonatal hypoglycaemia Micropenis Facial asymmetry SOD Kallman syndrome
	*GPR161*	AR	GHD	PSIS APH EPP Other: Emptysella	Alopecia Short 5th finger Ptosis left eye
**Other Syndromes**	*IGSF1*	X linked	TSH, GH, PRL deficiencies	/	Macroorchidism Delayedadrenarche
	*NFKB2*	AD	ACTH, TSH, GH deficiencies	/	Variable Immune deficiency (DAVID Syndrome)
	*PITX2*	AD	LH, FSH deficiencies GH insufficiency	APH Hypoplasia sellaturcica	Axenfeld—Rieger Syndrome: Anterior eye chamber Dental hypoplasia Craniofacial dysmorphism Protuberantumbilicus
	*CHD7*	AD	GH, TSH, FSH, LH deficiencies	EPP	CHARGE Syndrome
	*LHX3*	AR	CPHD (GH, TSH, FSH, LH, PRL, deficiencies variable ACTH deficiency)	Pituitary hypo- or hyperplasia Normal PP and stalk	Skeletal abnormalities Abnormal head and neck rotation (70%) Vertebral abnormalities—short cervical spine (50%) Sensorineural deafness (mild tosevere)
	*LHX4*	AD	Isolated GHD CPHD (GH, TSH, ACTH deficiencies, variable FSH, LH deficiencies)	APH PP normal or EPP Small sella turcica Corpus callosum hypoplasia Cerebellar abnormalities Chiari Syndrome	Lung defects—respiratory distress

*ACC, Agenesis corpus callosum; ACTH, Adrenocorticotropic hormone; AD, Autosomal Dominant; APH, Anterior Pituitary Hypoplasia; AR, Autosomal Recessive; CPHD, Combined Pituitary Hormone Deficiencies; EPP, Ectopic Posterior Pituitary; FSH, Follicle-stimulating hormone; GH, Growth Hormone; GHD, Growth Hormone deficiency; LH, Luteinizing Hormone; ONH, Optic Nerve Hypoplasia; PP, Posterior Pituitary; PRL, prolactin; PSIS, pituitary stalk interruption syndrome; SOD, Septo-optic Dysplasia; TSH, thyroid stimulatinghormone*.

**Table 2:** Mutations and characteristics of genes involved in non-syndromic Hypopituitarism.

**Transcription factor**	**Inheritance**	**Hormone deficiencies**	**MRI**	**Phenotype**
*PIT1/POU1F1*	AD, AR	GH, PRL, and TSH deficiencies	APH or normal AP Normal PP Normal infundibulum No extra pituitary abnormalities	No extra- pituitary abnormalities TSH deficiency may present early or develop muchlater
*PROP 1*	AR	GH, TSH, PRL, LH, FSH deficiencies Evolving ACTH deficiency	APH, normal or enlarged AP (transient, may change over time) Normal PP Normals talk	No extra-pituitary abnormalities Variable time of onset and severity of pituitary deficiencies

*ACC, Agenesis corpus callosum; ACTH, Adrenocorticotropic hormone; AD, Autosomal Dominant; APH, Anterior Pituitary Hypoplasia; AR, Autosomal Recessive; CPHD, Combined Pituitary Hormone Deficiencies; EPP, Ectopic Posterior Pituitary; FSH, Follicle-stimulating hormone; GH, Growth Hormone; GHD, Growth Hormone deficiency; LH, Luteinizing Hormone; ONH, Optic Nerve Hypoplasia; PP, Posterior Pituitary; PRL, prolactin; PSIS, pituitary stalk interruption syndrome; SOD, Septo-optic Dysplasia; TSH, thyroid stimulatinghormone*.

Different gene mutations can result in the same phenotype and different phenotypes can be secondary to the same single genetic mutation. Therefore, the clinical phenotype and associated morphological findings are crucial in the investigation of the underlying genetic mutations of cases of congenital hypopituitarism.

### Syndromic Hypopituitarism

Syndromic forms of CH are mainly due to mutations in transcription factors implicated during early pituitary development as listed in Table 1 and described in detail below.

#### Septo-Optic Dysplasia and Its Variants

Septo-optic dysplasia (SOD; de Morsier Syndrome) is an extremely heterogeneous and complex disorder defined by the presence of at least 2 of the following: (i) optic nerve hypoplasia (ONH), (ii) midline abnormalities seen in brain and pituitary MRI [mainly agenesis of the corpus callosum (ACC) and absence of the septum pellucidum], (iii) pituitary hypoplasia with hypopituitarism. Its estimated prevalence is 1 in 10,000 live births (17–19).

To date, transcription factors, such as *HESX1, SOX2, SOX3*, and *OTX2*, are the most common genes implicated in the etiology of SOD. Genetic mutations implicated in Kallmann syndrome (KS), such as *KAL1, FGFR1, PROKR2*, and *FGF8*, have also been recently identified in patients with SOD (16, 20, 21).

##### HESX1

The transcription factor *HESX1* is a member of the paired-like class of homeodomain proteins, the initial activation of which may be dependent upon *LHX1* and *OTX2*. *Hesx1* is one of the earliest markers of the pituitary primordium and can be detected in the anterior forebrain from E7.5 to E8.5 and in the Rathke's pouch from E8.5 to E135 (22). From E12, it is rapidly downregulated and becomes undetectable by E13.5 (23).

*Hesx1* is a transcriptional repressor and its down-regulation activates other downstream genes such as *Prop1*, suggesting that both function as opposing transcription factors.

Targeted disruption of *Hesx1* in mice results in anophthalmia or microphthalmia and midline neurological defects (such as absent septum pellucidum and pituitary hypoplasia), reminiscent of SOD (24).

The first homozygous missense mutation reported in *HESX1* (p.R160C) was described in two siblings with SOD born to consanguineous parents who presented with ACC, ONH, a hypoplastic AP gland and complete panhypopituitarism (25–27).

Since then, several homozygous and heterozygous *HESX1* mutations have been described. There is no clear genotype-phenotype correlation, and clinical features range from idiopathic GHD to CPHD, associated in some cases with anomalies such as SOD and pituitary malformations (28–33). MRI findings are variable, including a hypoplastic or aplastic AP and an ectopic posterior pituitary (EPP).

The phenotype of those patients with heterozygous mutations in *HESX1* tends to be milder presenting with isolated GHD and an ectopic or undescended posterior PP, although midline forebrain abnormalities can also be seen.

The majority of the cases are sporadic and just around 1% of the patients with SOD present with genetic mutations in *HESX1* (34, 35). In some patients, the penetrance is variable, suggesting the impact of additional genetic, or environmental factors.

##### SOX2

*SOX2* is a transcription factor member of the SRY-related HMG box (SOX) family. It is expressed at 4.5–9 weeks of human pituitary development within Rathke's pouch and is maintained throughout AP development as well as in the diencephalon.

It is expressed throughout the developing central nervous system as well as in sensory placodes, inner ear, cochlea and in the developing lens, retina, and optic nerve (36–44).

*SOX2* is extremely important in the maintenance of pituitary progenitor cells and its differentiation into all hormone-producing lineages (45).

The pituitary phenotype associated with murine *Sox2* loss of function mutations usually includes GH, TSH, and gonadotrophin deficiencies (46).

Heterozygous *de novo* mutations in humans have been observed in several patients with hypogonadotropic hypogonadism, bilateral, often severe, anophthalmia/microphthalmia, small corpus callosum, hippocampal abnormalities, and variable mental retardation (47–51). Esophageal atresia has also been reported (52, 53). The pituitary phenotype occasionally includes GH deficiency (GHD).

Imaging of the hypothalamo-pituitary region can show morphological anomalies such as hippocampal abnormalities, hypoplasia of the corpus callosum, hypothalamic hamartoma, and pituitary enlargement that is reminiscent of tumors (54).

##### SOX3

*SOX3* is also a member of the SRY-related HMG box (SOX) family. It is located on the X chromosome (Xp27.1) and is expressed along the full length of the central nervous system including the brain and spinal cord. *SOX3* dosage is critical for normal hypothalamic-pituitary development and both under- and over- dosage of the gene can lead to hypopituitarism (4, 55, 56).

Male patients present with variable hypopituitarism (CPHD or idiopathic GHD) and infundibular hypoplasia, an ectopic/undescended posterior pituitary (PP) and abnormalities of the corpus callosum. Intellectual disability is also frequently reported in these patients (4, 57, 58). Patients with duplication of *SOX3* can present with GHD without other pituitary deficiencies (59). Loss of function polyalanine expansions and gene deletions are associated with hypopituitarism including GH, TSH, ACTH, and gonadotrophin deficiencies. In terms of neuroradiological features, AP hypoplasia, an absent pituitary stalk, and ectopic EPP are other findings associated with *SOX3* sequence variants or whole gene deletions/duplications. Persistence of the craniopharyngeal canal has been reported in association with a *SOX3* deletion (60).

##### OTX2

Orthodenticle homeobox 2 (*Otx2*) is a transcription factor gene involved in brain, eye, nose and ear development (61, 62). It is expressed from E10.5 to E14.5 in the ventral diencephalon, from E10.5 to E12.5 in Rathke's pouch, and then becomes undetectable at both sites from E16.5 (63).

OTX2 regulates various transcription factors implicated in brain, eye and pituitary development, including *RX1, PAX6, SIX3, LHX2, MITF, GBX2*, and *HESX1* in order to coordinate cell determination and differentiation.

As *OTX2* is essential in retinal development, many patients with *OTX2* mutations and pituitary hormone deficiencies also present with a variety of ocular abnormalities. In humans, heterozygous *OTX2* mutations or gene deletions have been implicated in the etiology of 2–3% of anophthalmia/microphthalmia syndromes (64–67).

There is no clear genotype-phenotype correlation, even among patients with the same mutation. The pituitary phenotype ranges from partial to complete GHD and brain MRI can show a normal or hypoplastic AP, normal or EPP, and Chiari malformation (68, 69). In those patients without ocular involvement, who have CPHD and a small AP with or without an undescended PP, mutations in *OTX2* have been rarely reported (70). One of these rare cases is the p.N233S mutation where patients may not exhibit an ocular phenotype (71).

##### PAX6

PAX6 is an early dorsal marker of early AP gland and its expression is required for somatotrope, lactotrope, and thyrotrope development. It is also an important regulator of eye development, and heterozygous mutations in humans cause congenital eye anomalies (72). Recently, *PAX6* mutations have been reported to be associated with impaired pituitary function (ACTH deficiency, hypogonadotropic hypogonadism, and GHD) (73–75).

##### BMP4

Bone morphogenetic protein 4 (Bmp4) is the first secreted molecule detected in the prospective infundibulum at E8.5. It is essential for Rathke's pouch formation and maintenance. It is expressed in the optic vehicle, in the diencephalic floor and in the medial ganglionic eminence and in developing limbs. A recent study that included patients with eye abnormalities identified *BMP4* mutations in a familial case of anophthalmia, retinal dystrophy, brain malformation, and poly/syndactyly (76). Deletions in *BMP4* were associated with bilateral anophthalmia/microphthalmia, in association with hypothyroidism, deafness, developmental delay, and cerebellar and pituitary abnormalities.

##### FGFR1

FGF receptor 1 (FGFR1), a tyrosine kinase receptor for FGF, is the most important receptor involved in FGF8 signaling. Mutations in *FGFR1* have previously been reported in patients with Kallmann syndrome; more recently, *FGFR1* variants have been associated with CPHD, absent corpus callosum, SOD, and midline defects (77, 78).

##### ARNT2

Aryl hydrocarbon receptor nuclear translocator 2 (ARNT2) belongs to the HLH-PAS (Per-ARNT-Sim homology) subfamily of transcription factors. *Arnt2* is find on the hypothalamus, eye (neural retina), and kidney and urinary tract in rodents, and this expression pattern recapitulates that observed in humans (76).

#### Holoprosencephaly: Gli2 and FGF8

GLI2, a mediator of SHH signaling, is expressed in the ventral diencephalon inducing BMP4 and FGF8 expression, and also in the oral ectoderm, inducing pituitary progenitors. *GLI2* mutations are associated with holoprosencephaly (HPE) or HPE-like features with craniofacial anomalies, pituitary abnormalities and polydactyly (79–81).

Fibroblast growth factor 8 (*Fgf8*) is a member of the FGF family of signaling molecules that are involved in pituitary organogenesis. It is expressed in the infundibulum at E9.5, 1 day after the expression of Bmp4 (20, 82) and is important in midbrain development.

*FGF8* mutations are associated with Kallmann syndrome and have more recently been described in association with recessive HPE, craniofacial defects, and hypothalamo-pituitary dysfunction (83).

#### Hypopituitarism With Spine Abnormalities: LHX3

Expression of the LIM homeobox 3 (*LhX3*) gene, a member of the LIM class of homeodomain proteins, is detected early during AP development at E9.5 (Ratkhe's pouch, ventral hindbrain, and spinal cord) and persists in the mature pituitary gland. It is one of the earliest markers implicated in the anterior and intermediate lobes development and its expression plays an important role for the formation of gonadotrophs, thyrotrophs, somatotrophs, and lactotrophs (84).

Mice with homozygous mutations of *Lhx3* die soon after birth as a result of pituitary aplasia whereas those with heterozygous mutations have no abnormalities (85).

In humans, 14 homozygous (86–94) or compound heterozygous *LHX3* mutations (10) and a heterozygous variant (95) have been reported to date.

Commonly, patients with *LHX3* mutations present with GH, TSH, and FSH/LH deficiencies while ACTH deficiency is reported in 50% of cases (94).

The phenotype varies depending on which part of the gene is affected. If the mutation affects the entire gene or protein, the LIM domains or the homeodomain, patients will present with syndromes involving the nervous and skeletal systems, whereas if the mutation affects the carboxyl terminus of LHX3 protein alone, only pituitary dysfunction will be present. LHX3 is also required for inner ear development.

Extra-pituitary phenotypes can include a short neck with abnormal head and neck rotation (70% of cases), vertebral abnormalities (50% of cases) including a rigid cervical spine, flattened lumbar vertebrae, thoracic kyphosis, and progressive scoliosis, and variable degrees of sensorineural hearing loss (50% of cases). Developmental delay or learning difficulties have also been reported in nearly 40% of the patients. Two of the reported patients also had respiratory distress. Heterozygous family members are largely unaffected, although a recent publication has described a mild limitation of neck movement in a heterozygous carrier (10).

MRI findings can vary from a normal MRI (10% of the cases) to aplasia or hypoplasia of the AP, a hypointensity suggestive of microadenoma, and enlargement with a hyperintense signal (91).

#### Hypopituitarism With Cerebellar Abnormalities: LHX4

Lhx4 is closely related to Lhx3 and is expressed in the developing brain and spinal cord (96). It is also detected during early stages (E9.5 in Rathke's pouch and E12.5 in the anterior part of the pituitary) and is subsequently found in the future anterior lobe, with a decrease in expression by E15.5.

The AP gland in patients with *LHX4* mutations is hypoplastic, containing all the differentiated cell types but in reduced numbers. Other brain abnormalities can also be present such as an EPP and a hypoplastic sella turcica as well as corpus callosum hypoplasia or Chiari syndrome (97–105).

In humans, the phenotype can range from isolated GHD to complete panhypopituitarism (106). Several sporadic or familial *LHX4* mutations have been reported to date. Of note, four patients also presented with respiratory distress (76, 103, 107) and one presented with a cardiac defect (76). Several of the variants are variably penetrant, although the underlying mechanism remains to be established.

A lethal neonatal phenotype (severe panhypopituitarism associated with anterior pituitary aplasia and EPP, mild facial hypoplasia, undescended testes, and severe respiratory distress) has been recently described, secondary to a homozygous mutation (107).

#### Pituitary Stalk Interruption Syndrome (PSIS)

PSIS is a congenital defect of the pituitary gland characterized by the triad of (i) a thin pituitary stalk, (ii) an EPP gland, and (iii) hypoplasia or aplasia of the AP gland identified by MRI. Patients with PSIS may present with either isolated GHD or CPDH (22). Genetic alterations in *HESX1, LHX4, OTX2, SOX*3, and *PROKR2* have been reported in patients with PSIS, amongst others.

PROKR2, a G protein-coupled receptor essential for proper neuronal migration and angiogenesis, is involved in sex development and olfactory bulb development in mice. In humans, patients with mutations in *PROKR2* can present with hypogonadotropic hypogonadism or Kallmann syndrome. More recently, mutations have been associated with variably penetrant hypopituitarism including SOD (108–112). As described previously (section Septo-optic dysplasia and its variants), *OTX2* gene defects were also reported in patients with no ocular abnormalities (71).

GPR161, an orphan member of the G protein–coupled receptor family, has also been recently identified in patients with PSIS. GPR161 is widely expressed in both mouse and human during the early stages of embryogenesis including the neural folds, the pituitary and the hypothalamus.

It is a key negative regulator of the SHH pathway, the pituitary target of which is GLI2. It has been suggested that gain-of-function mutations of *GPR161* could lead to abnormal pituitary development by repressing the SHH pathway (22).

A homozygous missense mutation p.L19Q in *GPR161* has been recently described in two female siblings with short stature due to GHD associated with AP hypoplasia and an empty sella with an EPP. They also had a short 5th finger, congenital alopecia, and ptosis of the left eye (113).

#### Central Hypothyroidism and Macroorchidism

*IGSF1* is located at Xq26 and is expressed in Rathke's pouch, in the pituitary gland (present in GH, prolactin and TSH-secreting cells), and testis.

*IGSF1* mutations (loss of function or deletions) cause an X-linked syndrome. Male patients with mutations in *IGSF1* present with a characteristic phenotype that consists of congenital central hypothyroidism, delayed puberty, and adult macroorchidism. PRL and/or GH deficiencies have also been reported in some cases (114, 115). Some female patients with heterozygous mutations in *IGFS1* present with central congenital hypothyrodism, PRL deficiency, and delayed puberty.

#### Deficient Anterior Pituitary Function With Variable Immune Deficiency (DAVID) Syndrome

*NFKB2* belongs to the NF-κB family, which consists of a collection of evolutionarily conserved transcription factors involved primarily in development including the anterior pituitary gland, immunity, and oncogenesis.

Patients with *NFKB2* mutation present with deficit in AP gland function and common variable immune deficiency, a novel disorder called DAVID syndrome (116). However, the precise mechanism underlying endocrine deficits remains largely unclear.

#### Axenfeld—Rieger Syndrome

*Pitx2*, a homeobox gene, is detected in the stomodeum at E8, Rathke's pouch at E10.5 and 2 days after in the anterior and intermediate lobes. Patients affected with *PITX2* mutations present with Axenfeld–Rieger Syndrome which is characterized by eye, craniofacial, dental, cardiac, and umbilical anomalies (117).

#### CHARGE Syndrome and Pituitary Deficiencies

CHARGE syndrome is rare autosomal dominant disorder that affects multiple organs. It is characterized by ocular coloboma, cardiac defects, choanal atresia, growth, and developmental delay and ear abnormalities. EPP and hypopituitarism in patients with CHARGE syndrome has been recently reported in association with two novel *CHD7* variants (118).

#### Isolated ACTH Deficiency

Congenital isolated ACTH deficiency is mainly due to recessive mutations in *TBX19* (formerly *TPIT*) which are responsible of approximately 65% of the cases of isolated ACTH deficiency diagnosed during the first month of life (119). Neonates present with severe hypoglycaemia that can result in seizures and prolonged cholestatic jaundice. Biochemically this is characterized by low basal ACTH and cortisol concentrations and a poor ACTH response to corticotropin releasing hormone (CRH). It is extremely important to diagnose as this can be a potential cause of death during the neonatal period if no replacement treatment is started (120).

### Non—syndromic Combined Pituitary Hormone Deficiencies

Mutations in *PROP1* and *POU1F1* constitute the main genetic cause found in patients with non-syndromic GHD or CPHD (Table 2).

#### Mutations in PROP1

*Prop1* (prophet of PIT-1), a member of the paired-like family of homeodomain transcription factors, is the earliest expressed pituitary-specific transcription factor. It is detected within Rathke's pouch by E10, peaks at E12 and it disappears by E15.5 (121).

*Prop1* can act as both a transcriptional repressor (for *Hesx1* expression) or a transcriptional activator for *Pou1f1* (121, 122). Mice with *Prop1* over expression present with delayed puberty as a consequence of a delay in the differentiation of gonadotrophs (123).

The most frequent genetic cause of CPHD are recessive mutations in *PROP1* (124–130). The most common of these is a 2 base pair deletion within exon 2, which results in a frameshift at codon 101 and introduction of a termination codon at position 109 (130).

Recessive *PROP1* mutations are associated with GH, TSH, PRL and gonadotrophin deficiencies which vary in onset and severity. ACTH deficiency usually occurs later. They vary in the time of onset and severity and therefore it is important to have ongoing clinical surveillance.

GHD and growth delay is usually present during the first years of life in patients with *PROP1* mutations, however, there is a case report of a patient who had normal growth and achieved a normal adult height without GH treatment (131).

Both TSH and gonadotropin deficiency can appear at birth or later in life (132–134). Some patients may present with micropenis and undescended testes at birth and some others with delayed puberty. Spontaneous puberty can also be seen (135–137).

The majority of the patients do not have ACTH or cortisol deficiency during the first years of life but this can evolve later and ongoing surveillance is therefore needed (135, 138).

MRI shows variable pituitary morphology. The commonest finding is a normal pituitary stalk and posterior lobe with a small or normal AP gland. An enlarged AP gland has also been described with posterior regression (137).

#### Mutations in POU1F1

*POU1F1*, a member of the POU family, is expressed later during the pituitary organogenesis (E14.5) and persists during adulthood (139). It plays a crucial role in the regulation of the genes encoding GH, PRL, and TSH-beta and the time of onset and severity also varies. Gonadotroph and corticotroph axes usually remain functional.

Patients tend to present first with GH and prolactin deficiencies during the first years of life whereas TSH deficiency tends to present later (140).

*POU1F1* mutations are mainly recessive. However, dominant mutations have been recently described being the most frequent p.R271W mutation (141).

MRI reveals a normal or small AP gland; The PP and infundibulum are normal and no midline abnormalities have been reported (140, 142).

## Clinical Presentation, Diagnosis, and Treatment

Given the crucial regulatory role of the pituitary gland, prompt recognition of those neonates at risk of CH is important, as a delay in replacement therapy can have devastating consequences. Identifying neonates with CH can be challenging because they often present with non-specific symptoms such as hypoglycaemia, prolonged jaundiced, poor weight gain, temperature dysregulation, electrolyte abnormalities, and haemodynamic instability (Table 3).

**Table 3:** Clinical presentation of hypopituitarism in a neonate.

**Symptom/sign**	**Pituitary hormone deficiency**
Poor feeding	GH, ACTH
Poor weight gain	GH, ACTH, DI
Jitteriness	GH, ACTH
Lethargy	GH, ACTH
Seizures	ACTH
Recurrent sepsis	ACTH
Apnoea	ACTH
Conjugated jaundice	ACTH
Prolonged unconjugated jaundice	TSH
Temperature instability	TSH
Respiratory difficulties	TSH
Polyuria	DI
Polydipsia	DI
Undescended testes	Gonadotropin
Micropenis	Gonadotropin, GH

Birth weight and length tend to be normal, although GHD can lead to a slight reduction in birth weight. The clinical presentation and its severity depend on the number of hormones affected. These patients can have associated genital abnormalities, eye malformations, and/or midline defects.

Neonates with ACTH deficiency can present with cholestasis during the first 2 weeks of life. To understand the association between cholestasis and ACTH deficiency it is important to remind that cortisol increases bile flow and therefore, its deficiency will cause abnormalities in the synthesis and transport of bile acid leading to cholestasis in some cases.

A rise in transaminase concentrations can be seen after 2–4 weeks but GGT remains normal.

Once cortisol replacement treatment is started, cholestasis tends to resolve in around 10 weeks' time. In those cases where a liver biopsy is performed due to a delay in the diagnosis of CH, this shows canalicular cholestasis, and histopathology reveals mild portal eosinophilic infiltration.

Investigations to diagnose CH include baseline pituitary function tests (+ dynamic tests if indicated) and brain MRI. Genetic testing should also be considered.

However, the sensitivity and specificity of laboratory tests are limited in the newborn, especially in premature infants due to hypothalamo-pituitary axis immaturity, and lack of normative values. Additionally, GH stimulation tests are contraindicated under the age of 1 year.

A high index of suspicion for CH and early treatment in these patients is vital to avoid clinical decompensation. Treatment involves the physiological replacement of the relevant hormone deficiencies and requires close lifelong monitoring.

Individual hormone deficiencies are discussed in detail in the following section.

### ACTH Deficiency

#### Clinical Presentation

Neonates can present with failure to thrive, severe hypoglycaemia and cholestasis.

#### Diagnosis

As neonates do not have a circadian rhythm [reported to be established at around 2 months of age (143), or after 6 months of age (142), morning cortisol concentrations are not useful in evaluating ACTH deficiency in this population. Although cortisol deficiency related hypoglycaemia is severe, low cortisol concentrations at the time of hypoglycaemia have low specificity for the diagnosis of adrenal insufficiency and therefore should not be the only test for the diagnosis of ACTH deficiency (144). Many patients require a dynamic assessment (ACTH stimulation test using tetracosactide hexaacetate). The dose of tetracosactide hexaacetate used to diagnose central adrenal insufficiency, the timing for collection of blood samples for cortisol measurement, and the cut-off peak cortisol concentration for both the low-dose and standard ACTH test are the subject of much controversy. Stimulated cortisol concentrations ≥18 mg/dL (497 nmol/L) are indicative of a normal hypothalamo-pituitary-adrenal axis (145).

#### Treatment

In preterm infants, daily cortisol production is known to be ~7 mg/m^2^/day on the fifth day and ~6 mg/m^2^/day in the second week. When a neonate is diagnosed with ACTH deficiency, treatment needs to be started immediately. The treatment of choice is hydrocortisone due to its less potent side effects in terms of growth and bone health compared to other glucocorticoids. The starting dose of hydrocortisone is 9–12 mg/m^2^/day in 3–4 divided doses. This dose is higher compared to older infants because neonates have greater cortisol secretion rates. The dose can then be titrated with age. Prior to discharge, education for families about sick day rules and emergency dosing is important. In event of illness or stress, hydrocortisone doses should be doubled or even tripled. In event of an emergency, poor tolerance of oral hydrocortisone, or a suspected adrenal crisis, intramuscular hydrocortisone must be administered. The dose is age-dependent (<1 year 25 mg, 1–5 years 25–50 mg, >5 years 100 mg) and oral glucose should also be given to correct any associated hypoglycaemia. Those patients that cannot tolerate oral hydrocortisone require admission for intravenous hydrocortisone (1–2 mg/kg every 4–6 h). One they are able to tolerate oral hydrocortisone this is commenced at triple or double maintenance dose, and gradually weaned to maintenance depending on clinical improvement.

It is also important to highlight that cortisol deficiency can mask DI as cortisol is needed for water excretion. DI may develop after starting treatment with hydrocortisone and therefore close monitoring of fluid balance and electrolytes is important after starting glucocorticoid therapy (145).

Novel treatments such as continuous subcutaneous hydrocortisone infusion therapy, which may be difficult in neonates due to limited subcutaneous fat for insertion of the cannula, and sustained release hydrocortisone preparations aimed at mimicking physiological cortisol secretion remain to be established as potential therapies (146).

### TSH Deficiency

#### Etiology

Defects in TRH or TSH signaling are responsible of isolated central congenital hypothyroidism. As mentioned before, the most frequent genetic cause of isolated central congenital hypothyroidism is *IGSF1* gene mutation (147). Less common causes include genetic defects in TSH production, that is, mutations in the TRH receptor or TSH-B subunit (112, 148). More recently, mutations in *TBL1X* have been described in association with TSH deficiency.

#### Clinical Presentation

Newborns with TSH deficiency can present with prolonged physiological jaundice and low energy levels/sleepiness. Other findings such as temperature dysregulation, umbilical hernia, dry skin, bradycardia, macroglossia, and constipation may also be present.

X-linked central hypothyroidism due to *IGSF1* mutation is also later associated with delayed puberty and adult macroorchidism (149).

#### Diagnosis

Thyroid hormone is critical for normal brain development within the first 3 years of life, and therefore a prompt diagnosis is essential so that treatment can be commenced rapidly. Central hypothyroidism is characterized by the biochemical picture of low free T4 and usually low TSH (although it can also be inappropriately normal or even slightly elevated).

#### Treatment

Levothyroxine (LT4) is the treatment of choice in newborns with TSH deficiency at a starting dose between 10 and 15 μg/kg/day (150). However, higher doses will be needed in newborns with cholestasis due to malabsorption. Iron, soy, calcium, and anticonvulsants can also affect LT4 absorption and thus should not be co-administered with them. LT4 should ideally be given on an empty stomach but this is not always practical in neonates and so may need to be given with a small amount of milk. LT4 solution or crushed tablets can be given with water, breastmilk or formula.

For those babies unable to tolerate enteral preparations, intravenous tri-iodothyronine (T3) is available. The recommended intravenous dose is 75% of the total oral LT4 dose (151).

Before starting treatment with LT4, it is extremely important to exclude cortisol deficiency. LT4 increases basal metabolic rate, enhancing cortisol clearance with the subsequent risk of precipitating an adrenal crisis.

#### Monitoring

T4 concentrations should be monitored every 2–4 weeks during initial period of dose tritration. Thereafter monitoring may reduce in frequency. The aim is to keep fT4 in the mid-upper half of the normal range (152). TSH is not useful for monitoring in these cases.

### Gonadotrophin Deficiency

#### Clinical Presentation

Males present with micropenis, with or without undescended testes. Micropenis refers to a stretched penile length of −2.5 SD from the mean value: <1.5 cm at gestational age 30 weeks, 2 cm at 34 weeks, and <2.5 cm in term babies. Development of female genitalia is not affected by hypogonadotropic hypogonadism (HH) as it is independent of hormone secretion.

#### Diagnosis

##### Males

Mini puberty (raised LH and FSH) is seen between 15 days and 6 months old. Testosterone concentrations increase with a peak in the 4–10th week and start to decrease around the 6th month. LH concentrations <0.8 IU/L and testosterone <30 ng/mL between day 5 and 6 months of life are suggestive of the diagnosis. When an hCG test is done to assess testosterone production, penile growth and testicular descent may ensue and need to be documented. There are scant normative data pertaining to hCG tests in the first years of life. However, a study performed in adolescent males suggested that a peak LH concentration <2.8 IU/L after GnRH stimulation, with a testosterone peak of <3.6 nmol/L after 3 days of hCG injections and <9.5 nmol/L after 3 weeks of hCG injections are highly suggestive of hypogonadotrophic hypogonadism (153).

##### Females

Mini puberty is seen between 15 days and 2 years of age. FSH concentrations < 0.1 IU/L between 15 days and 2 years of life are diagnostic of probable hypogonadotrophic hypogonadism.

#### Treatment

In newborn male infants, the aim of the treatment is to ensure normal testicular descent, improve penile length and maximize fertility potential. Early treatment is recommended, ideally between 1 and 6 months of age. Testosterone can be given via intramuscular injections or topical gel (153–156). Testosterone injections (cypionate or enanthate) are commenced at a recommended dose of 25 mg every 4 weeks for 3 months. This is followed by clinical evaluation of the stretched penile length. Topical gel containing 5-α Dihydrotestosterone (DHT) is also useful and the recommended starting dose is 1 application (10 mg) every day for 3 months (153). The carer who is applying the testosterone gel should wash hands soon after the administration with soap and water and if the career is a female, the use of gloves is recommended. Cryptorchidism increases the risk of testicular neoplasia and also reduces fertility potential, therefore surgical correction (orchidopexy) is recommended during the first 2 years of life, ideally by 18 months of age (153, 157, 158). Treatment with LH and FSH during the neonatal period still remains under investigation (158–160).

### GH Deficiency

Congenital isolated GH deficiency (GHD) has an incidence of 1 in 4,000–1 in 10,000 live births (33), and is the most commonly affected pituitary hormone in childhood.

#### Etiology

Most of the cases are sporadic but there are four genetic forms that account for 5–30% of cases (161, 162). Congenital isolated GHD can be secondary to genetic mutations in the genes encoding growth hormone (*GH1*) or the growth hormone releasing hormone receptor (*GHRHR*), or in the genes encoding transcription factors SOX3, HESX1, GLI2, OTX2, LHX3, LHX4, PROP1, and POU1F1 (4, 163). Mutations in *GH1* and *GHRHR* may also lead to severe early growth failure with hypoglycaemia. Biallelic mutations in *RNPC3* have also been recently described patients with severe IGHD and AP hypoplasia (23, 164).

#### Clinical Presentation

Key features of (GHD) include hypoglycaemia and micropenis. It is important to note that GHD does not significantly affect fetal growth, and therefore, affected newborns are usually of normal weight and length at birth, with subsequent post-natal growth failure.

#### Diagnosis

GH evaluation in a neonate differs from that in an older child. During the neonatal period GH concentrations are higher in the term neonate during the first week of life than throughout childhood but a rapid decrease is seen during the following weeks (165). In contrast, IGF-1 concentrations (stimulated by GH) cannot be used as a screening test in neonates as they remain low for at least the first 15–18 months of age (166). A random GH concentration of less than or equal to 5 ng/mL (5 mcg/L) during the first 7 days of life accompanied by other pituitary hormone deficiencies and/or the classical imaging triad (EPP with AP hypoplasia and an abnormal stalk) is sufficient to diagnose GHD (165). Binder et al. (167) suggested that a GH cut-off of 7 μg/L as measured on a neonatal screening card by a highly sensitive polyclonal ELISA gave 100% sensitivity and 98% specificity. GH stimulation tests are considered dangerous and are contraindicated during the neonatal period, and a low GH concentration at the time of hypoglycaemia in isolation is not enough to diagnose GHD.

#### Treatment

In the event of persisting hypoglycaemia, GH treatment can be commenced during the neonatal period with daily subcutaneous recombinant human GH (rhGH) injections in the evening to mimic physiological growth hormone release. The initial recommended dose is between 0.16 and 0.24 mg/kg per week (22–35 mcg/kg per day) (165). Lower doses (10–20 mcg/kg/day) can also lead to excellent responses at this age. GH treatment can contribute to hypoglycaemia recovery and may improve cholestasis during the neonatal period (168).

#### Monitoring

Subsequent dosing should be individualized by monitoring IGF-I concentrations (at least every 3 months at the beginning). Patients also should be monitored for hypothyroidism and adrenal insufficiency as GH treatment increases metabolism of thyroid hormone and cortisol and may unmask these conditions.

### PRL Deficiency

#### Etiology

Prolactin deficiency is usually due to *POU1F1, LHX3, OTX2*, and *IGSF-1* gene mutations. It is important to note that some medications can affect PRL concentrations such as dopamine, calcium channel blockers and ranitidine.

#### Clinical Presentation

Puerperal alactogenesis is the only specific physical finding.

#### Diagnosis

A random prolactin concentrations <31 ng/mL during the neonatal period supports a diagnosis of PRL deficiency, however, breast tissue should not be palpated prior to a blood sample being taken as the levels could be falsely elevated. Prolactin concentrations are often elevated in association with midline defects.

#### Treatment

There is no commercially available treatment for PRL deficiency.

### Diabetes Insipidus (DI)

#### Etiology

In most cases of neonatal DI, anatomical defects or autosomal dominant or recessive genetic causes are present. DI is also observed in cases with SOD, corpus callosum agenesis and HPE. Renal concentrating mechanism can also be affected by other factors such as neonatal diabetes, hypercalcaemia, hypokalaemia. It is also important to note that mannitol, dextrose, saline fluids, and imaging contrast mediums can produce osmotic diuresis and secondary polyuria.

#### Clinical Presentation

The clinical features include polyhydramnios, polyuria, weight loss, irritability, dehydration, and hypernatremia.

#### Diagnosis

Diagnosis during the neonatal period is challenging as the capacity to concentrate urine is not as efficient as in older children and a water deprivation test is not recommended. Polyuria in DI during the neonatal period is defined as >5 mls/kg/h. Urine osmolality <300 mOsm/kg with a paired serum osmolality >300 mOsm/kg is suggestive of the diagnosis. If the urine osmolality is >600 mOsm/kg, DI is unlikely. The vasopressin test is useful to distinguish between central (CDI) and nephrogenic forms of DI but this can be hazardous during the neonatal period.

#### Treatment

Fluid therapy alone, without DDAVP, is the recommended management during the neonatal period as it can maintain euvolaemia. However, when CDI is extremely severe, a neonate may not respond to fluid therapy alone and DDAVP might be needed. In some cases of severe CDI, a thiazide diuretic may also be used. DDAVP can result in rapid fluid retention, hyponatremia and secondary cerebral oedema or even death in this vulnerable cohort of patients. Over-treatment is more dangerous than under-treatment and this is why a low starting dose of DDAVP (e.g., 1 μg) is recommended. Close monitoring (electrolytes, paired plasma and urine osmolalities, weight, and clinical examination for signs of fluid retention) is crucial, and dose adjustment will depend on the response to treatment (169). It is important to ensure breakthrough urine output prior to the next dose of DDAVP, in order to avoid severe fluid retention and hyponatraemia. It must also be highlighted that DDAVP may need to be withheld in neonates with concomitant ACTH deficiency who are unable to tolerate or absorb hydrocortisone when unwell (e.g., if vomiting), until appropriate steroid cover is provided. This is because cortisol is required for free water excretion and ongoing therapy with DDAVP without appropriate steroid replacement puts the neonate at risk of water intoxication.

## Imaging: Brain and Pituitary MRI

MRI of the brain and pituitary gland is recommended in all patients with suspected or confirmed CH. Abnormal brain and pituitary MRI findings do correlate with the severity and evolution of the disease (170).

The pituitary gland in newborns tends to be convex showing high signal intensity on T1-weighted images. As discussed previously, patients with CH usually have abnormal MRI findings ranging from a small AP gland to severe hypoplastic pituitary gland with EPP or undescended PP and an interrupted or hypoplastic pituitary stalk. A “bright spot” identifies the PP gland, however it can be absent in 10% of healthy individuals (170).

Pituitary/brain MRI studies should include qualitative description and dimensions of the AP; location and size of the PP gland, description of the pituitary stalk and comments about extra pituitary structures such as the optic chiasm, septum pellucidum, and corpus callosum. In order to have a proper description, the best technique is 2-mm to 3-mm thick, high-resolution T1-weighted, and T2-weighted images in coronal and sagittal planes.

The majority of newborns with severe CH show an EPP, abnormal pituitary stalk, and/or AP hypoplasia on MRI. This triad is known as “Pituitary Stalk Interruption Syndrome” (PSIS). Patients with IGHD and PSIS need to be closely monitored for evolving endocrinopathies as they can progress to CPHD.

Other midline brain abnormalities (absent/hypoplastic corpus callosum, absent septum pellucidum, schizencephaly, heterotopia) and ONH may be associated (33, 171).

## Conclusion

CH can be a life-threatening condition. A high index of suspicion is required for its early identification and treatment. However, early diagnosis during the neonatal period is challenging due to the variable and non-specific presenting symptoms. Red flag symptoms of CH include hypoglycaemia at birth and a micropenis.

In neonates with confirmed or suspected CH, a brain MRI with pituitary views is essential to exclude structural abnormalities. Ophthalmological review is also recommended to evaluate the optic nerves as many cases can have associated ocular abnormalities.

CH is an evolving and lifelong condition and therefore neonates with CH will require long term follow-up in order to detect early evolving endocrinopathies and optimize treatment. For those cases with a positive genetic finding, counseling is recommended (172). Currently, genetic analysis is successful in identifying an aetiological basis only in around 20% of cases (173). However, rapid advances in next-generation sequencing technology will help and improve our understanding of the complex mechanisms involved in congenital hypopituitarism (174). This technological progress is likely to have a positive impact on the clinical care of patients in the future (175).

## Author Contributions

Edited by LB. Reviewed by HK and MD. All authors contributed to the article and approved the submitted version.

## Conflict of Interest

The authors declare that the research was conducted in the absence of any commercial or financial relationships that could be construed as a potential conflict of interest.
